# Supplementary material for: Ornithological and molecular evidence of a reproducing Hyalomma rufipes population under continental climate in Europe
Source: Front Vet Sci. 2023 Mar 22;10:1147186. doi: 10.3389/fvets.2023.1147186 (PMC10073722; doi:10.3389/fvets.2023.1147186)
Supplement: Supplementary Figure 1 — The average annual precipitation (https://www.met.hu/) and temperature (https://www.mozaweb.com/search?search_=_középhomérséklet) in January in Hungary, based on data from the Hungarian Meteorological Service (OMSZ). The site of the discovered Hyalomma rufipes population is marked with a star. [file Image_1.pdf]

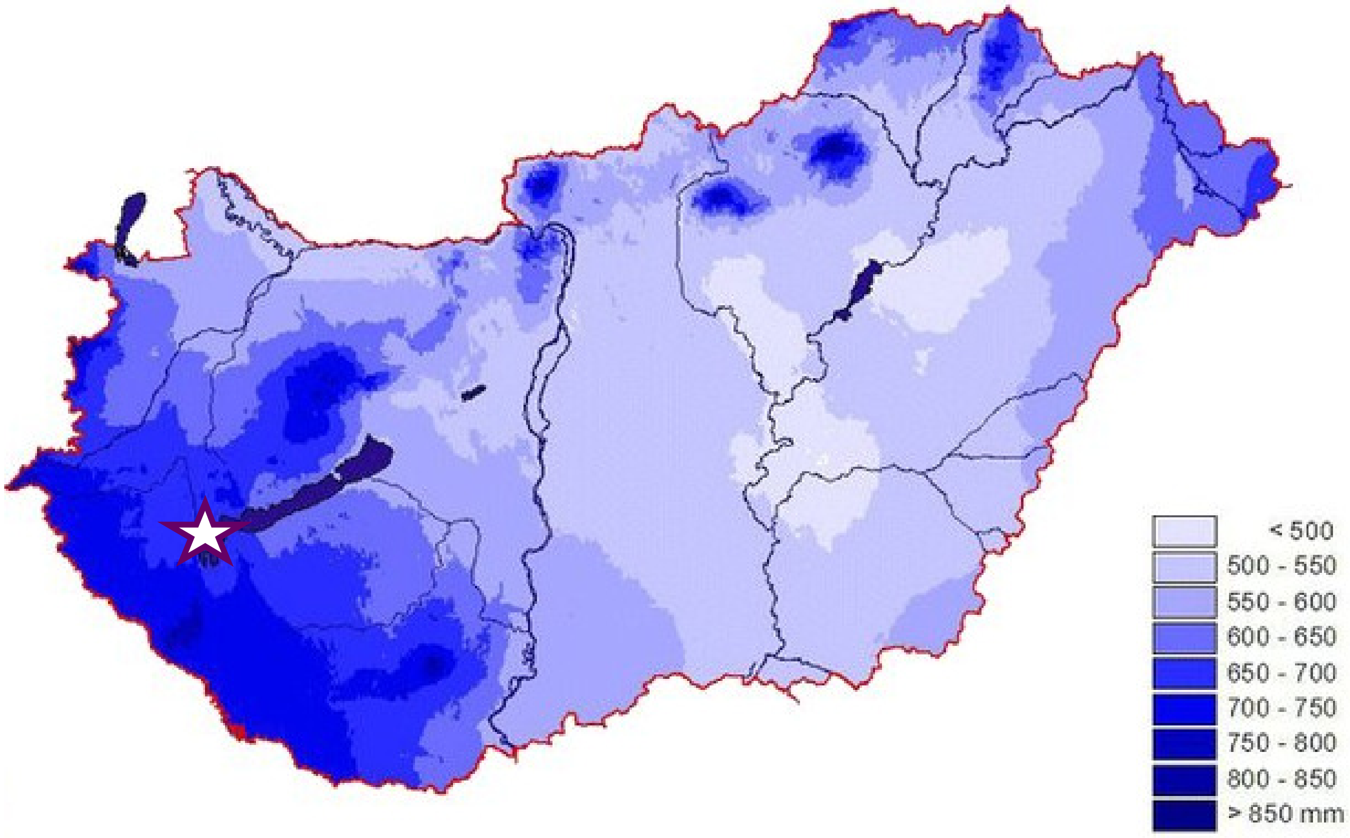

Average annual precipitation in Hungary (1971-2000).

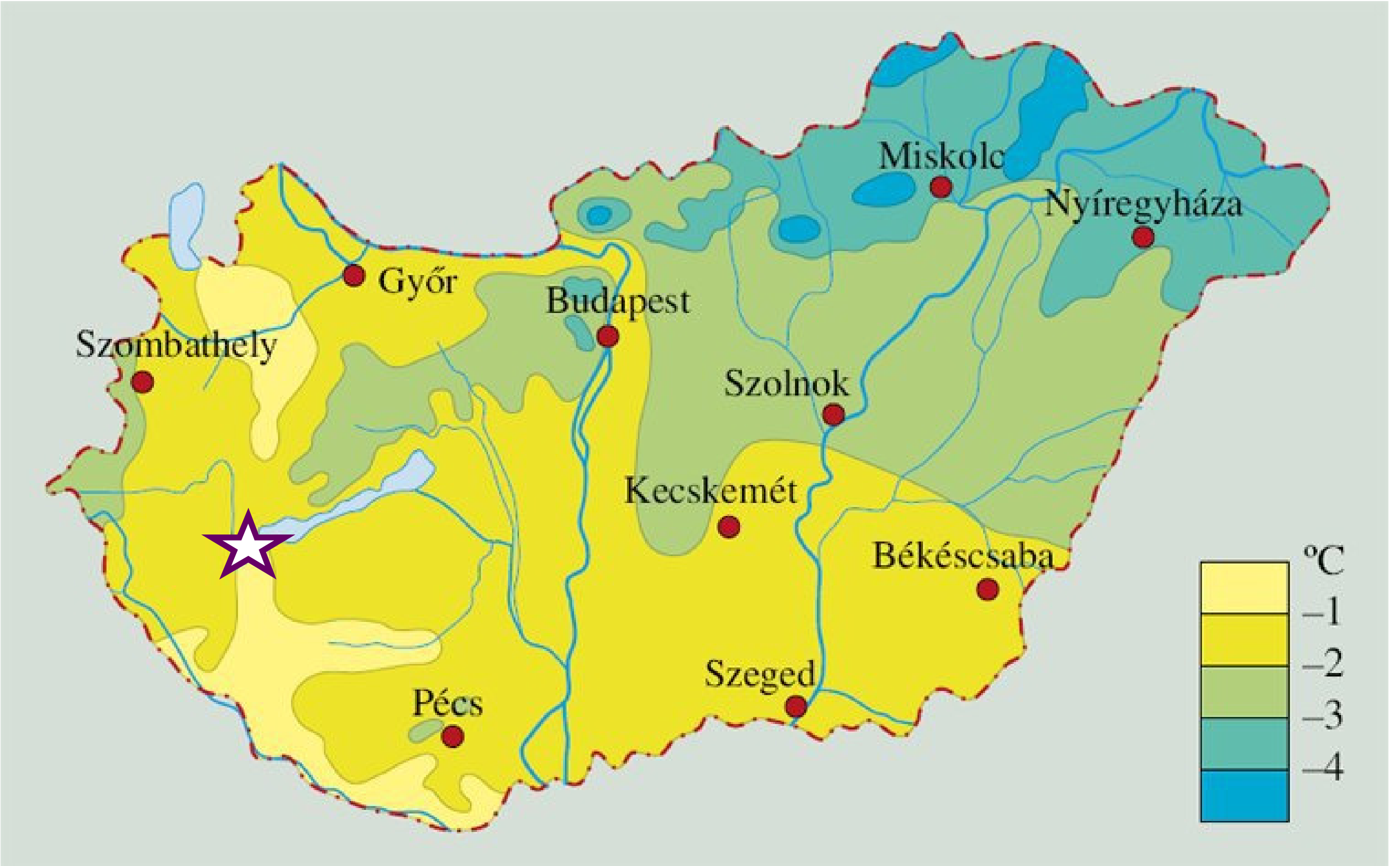

Average temperature in January in Hungary (data from the last two decades).
