# Supplementary material for: Ornithological and molecular evidence of a reproducing Hyalomma rufipes population under continental climate in Europe
Source: Front Vet Sci. 2023 Mar 22;10:1147186. doi: 10.3389/fvets.2023.1147186 (PMC10073722; doi:10.3389/fvets.2023.1147186)
Supplement: Supplementary Table 1 — Number of ticks for each bird ringing station, shown according to tick species, developmental stages, and collection periods. [file Table_1.pdf]

Supplementary Table 1

Location: Dávod

Spring migration and summer nesting period (March-July):

Tick species	Male	Female	Nymph	Larva	Total
I. ricinus	0	0	15	10	25
H. concinna	0	0	30	4	34
Hy. rufipes	0	0	1	0	1
I. frontalis	0	0	0	0	0
I. lividus	0	6	0	0	6

Autumn migration period (August-November):

Tick species	Male	Female	Nymph	Larva	Total
I. ricinus	0	0	7	3	10
H. concinna	0	0	6	1	7
Hy. rufipes	0	0	0	0	0
I. frontalis	0	0	0	0	0

Location: Lake Fehér

Spring migration and summer nesting period (March-July):

Tick species	Male	Female	Nymph	Larva	Total
I. ricinus	0	0	3	0	3
H. concinna	0	0	21	0	21
Hy. rufipes	0	0	0	0	0
I. frontalis	0	0	0	0	0

Autumn migration period (August-November):

Tick species	Male	Female	Nymph	Larva	Total
I. ricinus	0	0	0	1	1
H. concinna	0	0	11	3	14
Hy. rufipes	0	0	0	0	0
I. frontalis	0	0	0	0	0

Location: Fenékpuszta, Lake Balaton

Spring migration and summer nesting period (March-July):

Tick species	Male	Female	Nymph	Larva	Total
I. ricinus	0	0	4	0	4
H. concinna	0	0	20	1	21
Hy. rufipes	0	0	9	1	10
I. frontalis	0	0	0	0	0

Autumn migration period (August-November):

Tick species	Male	Female	Nymph	Larva	Total
I. ricinus	0	0	7	8	15
H. concinna	0	0	5	4	9
Hy. rufipes	0	0	0	0	0
I. frontalis	0	0	0	0	0

Location: **Izsák, Lake Kolon**

Spring migration and summer nesting period (March-July):

Tick species	Male	Female	Nymph	Larva	Total
I. ricinus	0	0	5	3	8
H. concinna	0	0	45	20	65
Hy. rufipes	0	0	0	0	0
I. frontalis	0	1	0	0	1

Autumn migration period (August-November):

Tick species	Male	Female	Nymph	Larva	Total
I. ricinus	0	0	9	2	11
H. concinna	0	0	8	7	15
Hy. rufipes	0	0	0	0	0
I. frontalis	0	2	0	0	2

Location: **Ócsa**

Spring migration and summer nesting period (March-July):

Tick species	Male	Female	Nymph	Larva	Total
I. ricinus	0	0	121	2	123
H. concinna	0	0	62	55	117
Hy. rufipes	0	0	0	0	0
I. frontalis	0	0	1	0	1
D. reticulatus	0	1	0	0	1

Autumn migration period (August-November):

Tick species	Male	Female	Nymph	Larva	Total
I. ricinus	0	0	50	34	84
H. concinna	0	0	5	3	8
Hy. rufipes	0	0	0	0	0
I. frontalis	0	1	1	3	5

Location: Bódva Valley

Spring migration and summer nesting period (March-July):

Tick species	Male	Female	Nymph	Larva	Total
I. ricinus	0	0	0	0	0
H. concinna	0	0	0	0	0
Hy. rufipes	0	0	0	0	0
I. frontalis	0	0	0	0	0

Autumn migration period (August-November):

Tick species	Male	Female	Nymph	Larva	Total
I. ricinus	0	0	50	149	199
H. concinna	0	0	1	2	3
Hy. rufipes	0	0	0	0	0
I. frontalis	0	0	2	0	2

Location: Tömörd

Spring migration and summer nesting period (March-July):

Tick species	Male	Female	Nymph	Larva	Total
I. ricinus	0	0	42	13	55
H. concinna	0	0	1	5	6
Hy. rufipes	0	0	1	0	1
I. frontalis	0	1	0	1	2

Autumn migration period (August-November):

Tick species	Male	Female	Nymph	Larva	Total
I. ricinus	0	0	44	16	60
H. concinna	0	0	1	0	1
Hy. rufipes	0	0	0	0	0
I. frontalis	0	4	0	1	5
