# Supplementary material for: Ornithological and molecular evidence of a reproducing Hyalomma rufipes population under continental climate in Europe
Source: Front Vet Sci. 2023 Mar 22;10:1147186. doi: 10.3389/fvets.2023.1147186 (PMC10073722; doi:10.3389/fvets.2023.1147186)
Supplement: Supplementary Table 2 — Avian host species that were found tick-infested in this study, shown according to collection site and spring or autumn migration intervals (the former including nesting period). [file Table_2.pdf]

Supplementary Table 2. Avian host species that were found tick-infested in this study, shown according to collection site and spring or autumn migration intervals (the former including nesting period). The number of tick-infested birds sampled at the same location is shown in parentheses after the abbreviation of bird species name. **Color code**: **red** - long distance migrant, **purple** - resident or short/mid-distance migrant, **blue** - resident.

Spring migration and nesting (March to July):

Tick species	Name of collection site						
	**Tömörd**	**Ócsa**	**Bódva Valley**	**Fenékpuszta**	**Baja, Dávod**	**Lake Kolon**	**Lake Fehér**
I. ricinus	ERI RUB (8)<br>TUR MER (6)<br>TUR PHI (3)<br>PAR CAE (2)<br>PAR MAJ (5)<br>PAR PAL (1)<br>ACR RIS (1)	TUR MER (13)<br>PAR MAJ (4)<br>COC COC (1)<br>TUR PHI (7)<br>ERI RUB (6)<br>TRO TRO (4)<br>ACR SCH (2)<br>SYL ATR (4)<br>SYL COM (1)<br>ACR SCI (4)<br>PHY COL (2)<br>LUS MEG (3)<br>LOC LUS (4)<br>SIT EUR (1)<br>ACR MEL (1)<br>ACR RIS (4)	-	ACR SCI (3)<br>ACR ARU (1)	EMB SCH (2)<br>PAN BIA (1)<br>ACR SCI (6)<br>ACR ARU (1)<br>TUR MER (1)<br>TUR PHI (1)<br>SYL ATR (1)<br>LUS MEG (1)<br>ACR RIS (3)	ACR SCH (1)<br>ACR SCI (2)<br>ACR RIS (2)<br>PAR MAJ (1)<br>LUS MEG (1)	ACR SCI (1)<br>ACR RIS (1)<br>HYP ICT (1)
H. concinna	ERI RUB (3)<br>TUR PHI (1)	TUR MER (1)<br>TUR PHI (2)<br>ACR SCH (6)<br>ACR SCI (2)<br>LUS MEG (1)<br>LOC LUS (17)<br>ACR MEL (2)<br>ACR RIS (4)	-	ACR SCI (5)<br>ACR SCH (7)<br>EMB SCH (1)<br>LOC LUS (4)	ACR SCI (6)<br>ACR ARU (1)<br>ACR MEL (1)<br>TUR MER (1)<br>TUR PHI (1)<br>SYL ATR (1)<br>LOC LUS (11)<br>ACR SCH (3)<br>LOC NAE (1)	ACR SCH (25)<br>LOC LUS (17)<br>ACR SCI (4)<br>ACR RIS (4)<br>LOC NAE (2)<br>PAR CAE (1)<br>LOC FLU (1)<br>ACR ARU (1)	ACR SCI (3)<br>LOC LUS (6)<br>ACR SCH (3)
Hyalomma sp.	FIC HYP (1)	-	-	ACR SCH (3)<br>PAN BIA (2)	SYL COM (1)	-	-
I. frontalis	ERI RUB (2)	ACR SCI (1)	-	-	-	ACR SCI (1)	-

Autumn migration (August to November):

Tick species	Name of collection site						
	Tömörd	Ócsa	River Bódva	Fenékpuszta	Baja, Dávod	Lake Kolon	Lake Fehér
I. ricinus	ERI RUB (7)<br>TUR MER (17)<br>TUR PHI (1)<br>PAR MAJ (1)<br>ANT TRI (1)<br>LUS MEG (1)<br>SYL ATR (4)<br>PHY TRO (1)<br>SYL COM (2)<br>PAS MON (2)<br>PHO OCH (1)<br>CAR CHL (1)<br>TUR ILI (1)<br>TRO TRO (2)	TUR MER (7)<br>PAR MAJ (1)<br>TUR PHI (2)<br>ERI RUB (16)<br>ACR SCH (1)<br>SYL ATR (8)<br>SYL COM (5)<br>ACR SCI (1)<br>LUS MEG (5)<br>ACR RIS (3)<br>SYL BOR (2)<br>LUS LUS (7)<br>CER BRA (1)<br>FRI COE (1)	ERI RUB (62)<br>SYL ATR (8)<br>SYL COM (6)<br>LUS MEG (1)<br>LUS LUS (1)<br>TUR MER (11)<br>FRI COE (1)<br>TUR PHI (2)<br>PHY TRO (1)	ACR SCI (2)<br>ACR SCH (2)<br>SYL ATR (2)<br>SYL COM (2)<br>HIR RUS (1)<br>LUS LUS (1)<br>PHY TRO (1)<br>LUS SVE (1)<br>ERI RUB (2)	SYL COM (1)<br>ACR SCI (1)<br>TUR MER (2)<br>SYL ATR (1)<br>ERI RUB (1)<br>TRO TRO (2)<br>ACR RIS (2)	LOC LUS (1)<br>LUS LUS (2)<br>ERI RUB (3)<br>TUR MER (1)	ACR RIS (1)
H. concinna	TUR MER (1)	TUR PHI (1)<br>ACR SCI (3)<br>LUS MEG (1)<br>LOC LUS (1)	ERI RUB (1)<br>SYL ATR (2)	ACR SCI (2)<br>ACR SCH (4)<br>ACR ARU (1)<br>LOC FLU (1)	ACR SCI (1)<br>TUR MER (1)<br>LOC LUS (3)<br>ACR SCH (1)<br>ACR RIS (1)	ACR ARU (1)<br>LOC LUS (5)<br>ACR RIS (1)<br>LOC NAE (1)	ACR SCI (1)<br>LOC LUS (1)<br>ACR ARU (2)
Hyalomma sp.	-	-	-	-	-	-	-
I. frontalis	ERI RUB (1)<br>TUR MER (2)<br>PAS MON (1)	TUR PHI (1)<br>ERI RUB (1)<br>PAS MON (1)	ERI RUB (1)<br>TUR MER (1)	-	-	ACR SCI (1)<br>TUR MER (1)	-

Abbreviations:

ACR RIS = *Acrocephalus palustris*, ACR SCH = *A. schoenobaenus*, ACR SCI = *A. scirpaceus*, LOC LUS = *Locustella luscinioides*, LOC NAE = *L. naevia*, PHY COL = *Phylloscopus collybita*, SYL ATR = *Sylvia atricapilla*, CAR CHL = *Carduelis chloris*, COC COC = *Coccothraustes coccothraustes*, EMB CIT = *Emberiza citrinella*, EMB SCH = *E. schoeniclus*, PAR MAJ = *Parus major*, LUS LUS = *Luscinia luscinia*, LUS MEG = *L. megarhynchos*, SYL COM = *S. communis*, TUR ILI = *Turdus iliacus*, ERI RUB = *Erithacus rubecula*, PRU MOD = *Prunella modularis*, TRO TRO = *Troglodytes troglodytes*, TUR MER = *T. merula*, TUR PHI = *T. philomelos*
