# Supplementary material for: Ornithological and molecular evidence of a reproducing Hyalomma rufipes population under continental climate in Europe
Source: Front Vet Sci. 2023 Mar 22;10:1147186. doi: 10.3389/fvets.2023.1147186 (PMC10073722; doi:10.3389/fvets.2023.1147186)
Supplement: Supplementary Table 3 — List of all ticks found in this study, sorted by date, collection site, and host species. [file Table_3.pdf]

	A	B	C	D	E	F	G	H	I	J	K	L	M	N	O
1	**Supplementary Table 3.**														
2	**SAMPLE ID**	**DATE**	**PLACE**	**BIRD SPECIES**	**Iric L**	**Iric N**	**Ifro L**	**Ifro N**	**Ifro F**	**Iliv F**	**Haec L**	**Haec N**	**Hyr L**	**Hyr N**	**Dret F**
3	BE01	2022.05.01	Fenékpuszta	ACR SCI		1									
4	BE02	2022.06.26	Fenékpuszta	ACR SCH										1	
5	BE03	2022.06.26	Fenékpuszta	ACR SCH										1	
6	BE04	2022.06.26	Fenékpuszta	ACR SCH										1	
7	BE05	2022.06.26	Fenékpuszta	PAN BIA										1	
8	BE06	2022.06.26	Fenékpuszta	PAN BIA									1	5	
9	BE11	2022.06.01	Fenékpuszta	ACR SCI		1						1			
10	BE14	2022.06.26	Fenékpuszta	ACR SCH								1			
11	BE16	2022.07.12	Fenékpuszta	LOC LUS								2			
12	BE17	2022.07.12	Fenékpuszta	ACR SCI								1			
13	BE19	2022.07.20	Fenékpuszta	ACR SCI								1			
14	BE20	2022.07.12	Fenékpuszta	ACR SCH							1	1			
15	BE21	2022.07.20	Fenékpuszta	ACR SCI		1									
16	BE22	2022.07.20	Fenékpuszta	ACR SCH								1			
17	BE23	2022.07.20	Fenékpuszta	EMB SCH								1			
18	BE29	2022.07.23	Fenékpuszta	ACR ARU		1									
19	BE30	2022.07.24	Fenékpuszta	ACR SCH								1			
20	BE31	2022.07.24	Fenékpuszta	LOC LUS								1			
21	BE33	2022.07.25	Fenékpuszta	ACR SCH								1			
22	BE34	2022.07.25	Fenékpuszta	ACR SCI								1			
23	BE35	2022.07.25	Fenékpuszta	LOC LUS								3			
24	BE36	2022.07.26	Fenékpuszta	LOC LUS								1			
25	BE37	2022.07.26	Fenékpuszta	ACR SCH								1			
26	BE38	2022.07.27	Fenékpuszta	ACR SCI								1			
27	BE39	2022.07.27	Fenékpuszta	ACR SCH								1			
28	BE46	2022.08.05	Fenékpuszta	ACR SCH								1			
29	BE49	2022.08.15	Fenékpuszta	HIR RUS	1										
30	BE53	2022.08.18	Fenékpuszta	ACR ARU								1			
31	BE58	2022.08.24	Fenékpuszta	ACR SCH							1				
32	BE63	2022.08.25	Fenékpuszta	ACR SCH								1			
33	BE65	2022.08.25	Fenékpuszta	ACR SCI								1			
34	BE123	2022.08.28	Fenékpuszta	ACR SCH	1										
35	BE146	2022.08.30	Fenékpuszta	SYL COM	1										
36	BE148	2022.08.31	Fenékpuszta	ACR SCI	1										
37	BE155	2022.09.03	Fenékpuszta	ACR SCH		1						1			
38	BE159	2022.09.03	Fenékpuszta	LUS LUS		1									
39	BE161	2022.09.05	Fenékpuszta	PHY TRO	1										
40	BE162	2022.09.05	Fenékpuszta	LOC FLU							2				
41	BE165	2022.09.05	Fenékpuszta	SYL COM		1									
42	BE166	2022.09.06	Fenékpuszta	LUS SVE	1										
43	BE167	2022.09.06	Fenékpuszta	SYL ATR		1									
44	BE169	2022.09.08	Fenékpuszta	ACR SCI							1				
45	BE171	2022.09.11	Fenékpuszta	ACR SCI		1									
46	BE173	2022.09.12	Fenékpuszta	SYL ATR	2										
47	BE177	2022.09.24	Fenékpuszta	ERI RUB		1									
48	BE180	2022.10.21	Fenékpuszta	ERI RUB		1									
49	GJ1	2022.03.28	Tömörd	TUR MER	1	4									
50	GJ2	2022.03.28	Tömörd	ERI RUB					1						
51	GJ3	2022.03.28	Tömörd	ERI RUB			1								
52	GJ4	2022.03.28	Tömörd	TUR MER		4									
53	GJ5	2022.03.29	Tömörd	ERI RUB	1										
54	GJ6	2022.03.29	Tömörd	ERI RUB	1										
55	GJ7	2022.04.23	Tömörd	TUR PHI	1	2									
56	GJ8	2022.04.23	Tömörd	TUR MER		5									
57	GJ9	2022.04.23	Tömörd	PAR CAE	1										
58	GJ10	2022.04.23	Tömörd	FIC HYP										1	
59	GJ11	2022.04.23	Tömörd	PAR MAJ		3									
60	GJ13	2022.04.30	Tömörd	TUR PHI		5									
61	GJ14	2022.05.14	Tömörd	TUR MER	2										
62	GJ15	2022.05.14	Tömörd	TUR MER		3									
63	GJ16	2022.05.14	Tömörd	TUR MER		2									
64	GJ17	2022.06.18	Tömörd	PAR CAE		1									
65	GJ18	2022.06.25	Tömörd	ERI RUB	1	1									
66	GJ19	2022.06.25	Tömörd	ERI RUB	1										
67	GJ20	2022.06.25	Tömörd	ERI RUB		1					1				
68	GJ21	2022.06.25	Tömörd	PAR MAJ		1									
69	GJ22	2022.06.25	Tömörd	PAR MAJ		2									
70	GJ23	2022.06.25	Tömörd	ERI RUB	3						3				
71	GJ24	2022.06.25	Tömörd	ERI RUB		1									
72	GJ25	2022.06.25	Tömörd	PAR MAJ		2									
73	GJ26	2022.06.25	Tömörd	PAR MAJ		1									
74	GJ27	2022.06.25	Tömörd	TUR PHI		1						1			
75	GJ28	2022.06.25	Tömörd	ERI RUB							1				
76	GJ29	2022.06.25	Tömörd	ERI RUB	1	1									
77	GJ30	2022.07.31	Tömörd	PAR PAL		1									
78	GJ31	2022.07.31	Tömörd	ACR RIS		1									
79	GJ32	2022.08.04	Tömörd	ANT TRI	3										
80	GJ33	2022.08.04	Tömörd	LUS MEG	1										
81	GJ34	2022.08.05	Tömörd	SYL ATR	1	1									
82	GJ35	2022.08.05	Tömörd	TUR MER								1			
83	GJ36	2022.08.05	Tömörd	PAR MAJ	1										
84	GJ37	2022.08.08	Tömörd	TUR MER		2									
85	GJ39	2022.08.10	Tömörd	PHY TRO		1									
86	GJ40	2022.08.14	Tömörd	SYL COM		1									
87	GJ41	2022.09.05	Tömörd	PAS MON		1									
88	GJ42	2022.09.05	Tömörd	PAS MON					2						
89	GJ43	2022.09.06	Tömörd	ERI RUB	1										
90	GJ44	2022.09.06	Tömörd	TUR MER		3									
91	GJ45	2022.09.13	Tömörd	SYL ATR		1									
92	GJ46	2022.09.13	Tömörd	SYL COM		1									
93	GJ47	2022.09.13	Tömörd	TUR MER		1									
94	GJ48	2022.09.13	Tömörd	ERI RUB	1										
95	GJ49	2022.09.04	Tömörd	TUR MER					1						
96	GJ50	2022.10.01	Tömörd	ERI RUB		1									
97	GJ51	2022.10.02	Tömörd	TUR MER		2									
98	GJ52	2022.10.02	Tömörd	PHO OCH		1									
99	GJ53	2022.10.02	Tömörd	TUR MER		1									
100	GJ54	2022.10.02	Tömörd	TUR MER		1									

	A	B	C	D	E	F	G	H	I	J	K	L	M	N	O
101	GJ55	2022.10.02	Tömörd	SYL ATR		1									
102	GJ56	2022.10.07	Tömörd	ERI RUB		2									
103	GJ58	2022.10.08	Tömörd	TUR MER		2									
104	GJ59	2022.10.10	Tömörd	ERI RUB	1										
105	GJ60	2022.10.10	Tömörd	TUR MER		1									
106	GJ61	2022.10.10	Tömörd	ERI RUB		1									
107	GJ62	2022.10.10	Tömörd	TUR MER		1									
108	GJ63	2022.10.10	Tömörd	TUR MER		1									
109	GJ64	2022.10.10	Tömörd	TUR MER		1									
110	GJ65	2022.10.10	Tömörd	TUR MER		1									
111	GJ66	2022.10.10	Tömörd	CAR CHL		1									
112	GJ67	2022.10.10	Tömörd	TUR MER	1	1									
113	GJ68	2022.10.10	Tömörd	PAS MON		1									
114	GJ69	2022.10.10	Tömörd	SYL ATR	1										
115	GJ70	2022.10.11	Tömörd	TUR PHI	3										
116	GJ71	2022.10.11	Tömörd	ERI RUB		1									
117	GJ72	2022.10.13	Tömörd	TUR MER		1									
118	GJ73	2022.10.13	Tömörd	TUR MER		3									
119	GJ74	2022.10.13	Tömörd	TUR MER	1										
120	GJ75	2022.10.13	Tömörd	TUR ILI		1									
121	GJ76	2022.10.22	Tömörd	TRO TRO		3									
122	GJ77	2022.10.22	Tömörd	TUR MER					1						
123	GJ78	2022.10.25	Tömörd	TRO TRO	1										
124	GJ79	2022.10.29	Tömörd	TUR MER		3									
125	GJ80	2022.10.29	Tömörd	ERI RUB			1								
126	HA1	2022.08.16	Bódva Valley	ERI RUB		1									
127	HA2	2022.08.16	Bódva Valley	ERI RUB	3										
128	HA3	2022.08.16	Bódva Valley	SYL ATR	3										
129	HA4	2022.08.16	Bódva Valley	ERI RUB								1			
130	HA5	2022.08.17	Bódva Valley	ERI RUB	7										
131	HA6	2022.08.17	Bódva Valley	SYL COM		1									
132	HA7	2022.08.18	Bódva Valley	ERI RUB	1										
133	HA8	2022.08.18	Bódva Valley	ERI RUB	30										
134	HA9	2022.08.18	Bódva Valley	ERI RUB	3										
135	HA10	2022.08.18	Bódva Valley	LUS MEG	5										
136	HA11	2022.08.19	Bódva Valley	LUS LUS	3										
137	HA12	2022.08.19	Bódva Valley	ERI RUB	4										
138	HA13	2022.08.20	Bódva Valley	ERI RUB	4										
139	HA14	2022.08.22	Bódva Valley	TUR MER	1										
140	HA15	2022.08.22	Bódva Valley	TUR MER		1									
141	HA16	2022.08.22	Bódva Valley	ERI RUB	1										
142	HA17	2022.08.22	Bódva Valley	ERI RUB	2										
143	HA18	2022.08.22	Bódva Valley	SYL ATR	1										
144	HA19	2022.08.22	Bódva Valley	ERI RUB	2										
145	HA20	2022.08.23	Bódva Valley	ERI RUB	1										
146	HA21	2022.08.24	Bódva Valley	ERI RUB	1										
147	HA22	2022.08.25	Bódva Valley	ERI RUB	1	1									
148	HA23	2022.08.25	Bódva Valley	SYL ATR							1				
149	HA24	2022.08.25	Bódva Valley	ERI RUB	1										
150	HA25	2022.08.25	Bódva Valley	ERI RUB	1										
151	HA26	2022.08.26	Bódva Valley	ERI RUB	1										
152	HA27	2022.08.26	Bódva Valley	SYL COM		1									
153	HA28	2022.08.26	Bódva Valley	SYL ATR							1				
154	HA30	2022.08.27	Bódva Valley	ERI RUB	2										
155	HA31	2022.08.27	Bódva Valley	SYL COM		1									
156	HA32	2022.08.27	Bódva Valley	ERI RUB		1									
157	HA33	2022.08.28	Bódva Valley	TUR MER		1									
158	HA34	2022.08.28	Bódva Valley	TUR MER		2		1							
159	HA35	2022.09.06	Bódva Valley	SYL ATR		1									
160	HA36	2022.09.06	Bódva Valley	SYL COM		1									
161	HA37	2022.09.07	Bódva Valley	SYL COM		1									
162	HA38	2022.09.08	Bódva Valley	SYL COM	1										
163	HA39	2022.09.09	Bódva Valley	SYL ATR	1										
164	HA40	2022.09.12	Bódva Valley	ERI RUB	1										
165	HA41	2022.09.12	Bódva Valley	SYL ATR	1	2									
166	HA42	2022.09.12	Bódva Valley	ERI RUB	1										
167	HA43	2022.09.12	Bódva Valley	TUR MER		1									
168	HA44	2022.09.12	Bódva Valley	FRI COE	1										
169	HA45	2022.09.12	Bódva Valley	TUR MER		1									
170	HA46	2022.09.12	Bódva Valley	ERI RUB	3										
171	HA47	2022.09.13	Bódva Valley	SYL ATR		1									
172	HA48	2022.09.13	Bódva Valley	ERI RUB	1										
173	HA49	2022.09.13	Bódva Valley	ERI RUB	1										
174	HA50	2022.09.13	Bódva Valley	ERI RUB		1									
175	HA51	2022.09.13	Bódva Valley	ERI RUB	1										
176	HA52	2022.09.13	Bódva Valley	SYL ATR		1									
177	HA53	2022.09.13	Bódva Valley	ERI RUB		1									
178	HA54	2022.09.13	Bódva Valley	TUR MER		2									
179	HA55	2022.09.13	Bódva Valley	ERI RUB				1							
180	HA56	2022.09.14	Bódva Valley	ERI RUB	1										
181	HA57	2022.09.14	Bódva Valley	ERI RUB	1										
182	HA58	2022.09.14	Bódva Valley	ERI RUB		1									
183	HA59	2022.09.14	Bódva Valley	ERI RUB	1										
184	HA60	2022.09.14	Bódva Valley	ERI RUB	2										
185	HA61	2022.09.15	Bódva Valley	ERI RUB	1	1									
186	HA62	2022.09.15	Bódva Valley	ERI RUB		1									
187	HA63	2022.09.16	Bódva Valley	TUR PHI	7	2									
188	HA64	2022.09.16	Bódva Valley	TUR MER		3									
189	HA65	2022.09.16	Bódva Valley	TUR MER	3										
190	HA66	2022.09.16	Bódva Valley	TUR MER	4	4									
191	HA68	2022.09.16	Bódva Valley	ERI RUB		1									
192	HA69	2022.09.16	Bódva Valley	ERI RUB	1										
193	HA71	2022.09.16	Bódva Valley	PHY TRO		1									
194	HA72	2022.09.16	Bódva Valley	ERI RUB	3	1									
195	HA73	2022.09.16	Bódva Valley	ERI RUB	2										
196	HA74	2022.09.16	Bódva Valley	ERI RUB		1									
197	HA75	2022.09.16	Bódva Valley	ERI RUB	1										
198	HA76	2022.09.16	Bódva Valley	ERI RUB	2										
199	HA77	2022.09.16	Bódva Valley	ERI RUB		1									
200	HA78	2022.09.16	Bódva Valley	TUR MER	1	1									

	A	B	C	D	E	F	G	H	I	J	K	L	M	N	O
201	HA79	2022.09.16	Bódva Valley	ERI RUB	2										
202	HA80	2022.09.16	Bódva Valley	ERI RUB	1										
203	HA81	2022.09.16	Bódva Valley	ERI RUB	4										
204	HA82	2022.09.16	Bódva Valley	ERI RUB	2										
205	HA83	2022.09.16	Bódva Valley	ERI RUB	1										
206	HA84	2022.09.16	Bódva Valley	ERI RUB	1	1									
207	HA85	2022.09.16	Bódva Valley	ERI RUB	2										
208	HA86	2022.09.16	Bódva Valley	ERI RUB	1										
209	HA87	2022.09.17	Bódva Valley	TUR PHI	2										
210	HA88	2022.09.17	Bódva Valley	ERI RUB	1										
211	HA89	2022.09.17	Bódva Valley	ERI RUB	1										
212	HA90	2022.09.17	Bódva Valley	ERI RUB		2									
213	HA91	2022.09.17	Bódva Valley	ERI RUB	1										
214	HA92	2022.09.17	Bódva Valley	ERI RUB	6										
215	HA93	2022.09.17	Bódva Valley	ERI RUB		1									
216	HA94	2022.09.17	Bódva Valley	ERI RUB	2										
217	HA95	2022.09.17	Bódva Valley	ERI RUB	1										
218	HA96	2022.09.17	Bódva Valley	ERI RUB		2									
219	HA97	2022.09.17	Bódva Valley	ERI RUB		1									
220	HA98	2022.09.17	Bódva Valley	SYL ATR		1									
221	HA99	2022.09.17	Bódva Valley	ERI RUB	1										
222	HA100	2022.09.17	Bódva Valley	ERI RUB		1									
223	KO1	2022.04.11	Lake Fehér	ACR SCI		1									
224	KO2	2022.06.25	Lake Fehér	LOC LUS								1			
225	KO3	2022.06.25	Lake Fehér	LOC LUS								2			
226	KO4	2022.06.25	Lake Fehér	LOC LUS								7			
227	KO5	2022.06.25	Lake Fehér	ACR SCI								2			
228	KO6	2022.07.18	Lake Fehér	ACR SCH								1			
229	KO7	2022.07.18	Lake Fehér	ACR SCH								1			
230	KO8	2022.07.18	Lake Fehér	LOC LUS								1			
231	KO9	2022.07.20	Lake Fehér	ACR SCI								2			
232	KO10	2022.07.20	Lake Fehér	ACR SCH								1			
233	KO11	2022.07.21	Lake Fehér	ACR RIS		1									
234	KO12	2022.07.22	Lake Fehér	LOC LUS								1			
235	KO13	2022.07.23	Lake Fehér	ACR SCI								1			
236	KO14	2022.07.23	Lake Fehér	HYP ICT		1									
237	KO15	2022.07.23	Lake Fehér	LOC LUS								1			
238	KO16	2022.08.02	Lake Fehér	ACR ARU								1			
239	KO17	2022.08.03	Lake Fehér	LOC LUS							3	8			
240	KO18	2022.08.03	Lake Fehér	ACR RIS	1										
241	KO19	2022.08.03	Lake Fehér	ACR SCI								1			
242	KO21	2022.08.16	Lake Fehér	ACR ARU								1			
243	BA1	2022.04.15	Dávod (and it	EMB SCH		1									
244	BA2	2022.05.14	Dávod (and it	SYL COM										1	
245	BA3	2022.05.27	Dávod (and it	ACR RIS		1									
246	BA4	2022.05.27	Dávod (and it	PAN BIA		1									
247	BA5	2022.06.04	Dávod (and it	ACR SCI		1									
248	BA6	2022.06.04	Dávod (and it	ACR ARU								1			
249	BA7	2022.06.04	Dávod (and it	ACR SCI		1									
250	BA8	2022.06.04	Dávod (and it	ACR MEL								1			
251	BA10	2022.06.19	Dávod (and it	ACR SCI		1									
252	BA11	2022.06.19	Dávod (and it	ACR ARU		1									
253	BA12	2022.06.19	Dávod (and it	TUR MER		2					1	3			
254	BA13	2022.06.19	Dávod (and it	TUR PHI								1			
255	BA14	2022.06.26	Dávod (and it	SYL ATR								1			
256	BA15	2022.06.26	Dávod (and it	ACR SCI								1			
257	BA17	2022.06.26	Dávod (and it	ACR SCH							1				
258	BA18	2022.07.01	Dávod (and it	RIP RIP						1					
259	BA19	2022.07.01	Dávod (and it	RIP RIP						1					
260	BA20	2022.07.01	Dávod (and it	RIP RIP						1					
261	BA21	2022.07.01	Dávod (and it	RIP RIP						1					
262	BA22	2022.07.03	Dávod (and it	RIP RIP						1					
263	BA23	2022.07.04	Dávod (and it	RIP RIP						1					
264	BA24	2022.07.05	Dávod (and it	ACR SCI								1			
265	BA25	2022.07.05	Dávod (and it	ACR SCI								1			
266	BA26	2022.07.05	Dávod (and it	LOC LUS								1			
267	BA27	2022.07.11	Dávod (and it	ACR SCI								1			
268	BA28	2022.07.11	Dávod (and it	ACR SCI		1									
269	BA29	2022.07.11	Dávod (and it	ACR SCI		2									
270	BA30	2022.07.12	Dávod (and it	LOC LUS								1			
271	BA31	2022.07.12	Dávod (and it	LOC LUS								2			
272	BA32	2022.07.13	Dávod (and it	LOC NAE							2				
273	BA33	2022.07.13	Dávod (and it	ACR SCI								1			
274	BA34	2022.07.14	Dávod (and it	ACR SCI	1										
275	BA35	2022.07.14	Dávod (and it	LOC LUS								2			
276	BA36	2022.07.16	Dávod (and it	ACR SCI								1			
277	BA37	2022.07.19	Dávod (and it	ACR SCH								1			
278	BA38	2022.07.22	Dávod (and it	LOC LUS								2			
279	BA39	2022.07.23	Dávod (and it	SYL ATR	1										
280	BA40	2022.07.25	Dávod (and it	ACR SCH								1			
281	BA41	2022.07.25	Dávod (and it	ACR RIS	7										
282	BA42	2022.07.26	Dávod (and it	LOC LUS								1			
283	BA43	2022.07.26	Dávod (and it	LOC LUS								1			
284	BA44	2022.07.26	Dávod (and it	LOC LUS								1			
285	BA45	2022.07.27	Dávod (and it	LOC LUS								1			
286	BA46	2022.07.27	Dávod (and it	LOC LUS								1			
287	BA47	2022.07.27	Dávod (and it	ACR RIS	1										
288	BA48	2022.07.27	Dávod (and it	EMB SCH		1									
289	BA49	2022.07.28	Dávod (and it	LOC LUS								2			
290	BA50	2022.08.01	Dávod (and it	ACR SCI		1									
291	BA51	2022.08.01	Dávod (and it	LOC LUS								1			
292	BA52	2022.08.03	Dávod (and it	SYL ATR	1										
293	BA53	2022.08.03	Dávod (and it	ACR SCI								1			
294	BA54	2022.08.04	Dávod (and it	ACR SCH								1			
295	BA55	2022.08.06	Dávod (and it	ACR RIS								1			
296	BA56	2022.08.10	Dávod (and it	ACR RIS	1										
297	BA57	2022.08.10	Dávod (and it	LOC LUS							1				
298	BA58	2022.08.12	Dávod (and it	LOC LUS								1			
299	BA59	2022.08.12	Dávod (and it	SYL COM		1									
300	BA60	2022.09.11	Dávod (and it	ACR RIS		1									

	A	B	C	D	E	F	G	H	I	J	K	L	M	N	O
301	BA61	2022.10.06	Dávod (and it	ERI RUB		1									
302	BA62	2022.10.17	Dávod (and it	TRO TRO		1									
303	BA63	2022.10.18	Dávod (and it	TRO TRO	1										
304	BA64	2022.10.18	Dávod (and it	TUR MER								1			
305	BA65	2022.11.07	Dávod (and it	TUR MER		2									
306	KB1	2022.05.19	Dávod (and it	LUS MEG		1									
307	KB2	2022.05.31	Dávod (and it	TUR PHI		1									
308	KB3	2022.07.13	Lake Kolon	ACR SCH								1			
309	KB4	2022.07.15	Lake Kolon	ACR ARU								1			
310	KB5	2022.07.17	Lake Kolon	ACR SCH								1			
311	KB6	2022.07.17	Lake Kolon	ACR SCH								1			
312	KB7	2022.07.17	Lake Kolon	ACR SCH								1			
313	KB8	2022.07.18	Lake Kolon	ACR RIS								1			
314	KB9	2022.07.23	Lake Kolon	LOC LUS								1			
315	KB10	2022.07.23	Lake Kolon	LOC LUS								1			
316	KB11	2022.07.23	Lake Kolon	ACR RIS							1	1			
317	NA1	2022.04.10	Lake Kolon	ACR SCI					1						
318	NA2	2022.04.14	Lake Kolon	PAR MAJ		1									
319	NA3	2022.04.14	Lake Kolon	ACR SCI		2									
320	NA4	2022.07.07	Lake Kolon	ACR SCH								1			
321	NA5	2022.07.11	Lake Kolon	LOC NAE								1			
322	NA6	2022.07.11	Lake Kolon	ACR SCH								1			
323	NA7	2022.07.13	Lake Kolon	ACR RIS								1			
324	NA8	2022.07.13	Lake Kolon	ACR RIS								1			
325	NA10	2022.07.13	Lake Kolon	ACR RIS	1										
326	NA12	2022.07.13	Lake Kolon	ACR RIS	1										
327	NA13	2022.07.13	Lake Kolon	ACR SCH		1									
328	NA14	2022.07.15	Lake Kolon	ACR SCH							1				
329	NA15	2022.07.15	Lake Kolon	LOC LUS								1			
330	NA16	2022.07.15	Lake Kolon	LOC LUS								1			
331	NA17	2022.07.15	Lake Kolon	LOC LUS								1			
332	NA18	2022.07.15	Lake Kolon	LOC LUS								1			
333	NA19	2022.07.15	Lake Kolon	LOC LUS								1			
334	NA21	2022.07.15	Lake Kolon	LOC LUS								1			
335	NA23	2022.07.15	Lake Kolon	LOC LUS								1			
336	NA24	2022.07.16	Lake Kolon	ACR SCH								1			
337	NA25	2022.07.16	Lake Kolon	LOC LUS								1			
338	NA26	2022.07.16	Lake Kolon	LOC LUS								1			
339	NA27	2022.07.16	Lake Kolon	LOC LUS								3			
340	NA28	2022.07.16	Lake Kolon	LOC LUS								2			
341	NA30	2022.07.17	Lake Kolon	LOC LUS								1			
342	NA31	2022.07.18	Lake Kolon	ACR SCH							1	1			
343	NA32	2022.07.18	Lake Kolon	ACR SCH							1				
344	NA33	2022.07.18	Lake Kolon	ACR SCH							1				
345	NA34	2022.07.18	Lake Kolon	ACR SCH							1				
346	NA35	2022.07.18	Lake Kolon	ACR SCH							1				
347	NA36	2022.07.18	Lake Kolon	ACR SCH							1				
348	NA37	2022.07.18	Lake Kolon	ACR SCH							1				
349	NA38	2022.07.18	Lake Kolon	ACR SCH							1				
350	NA39	2022.07.18	Lake Kolon	ACR SCH							1				
351	NA40	2022.07.18	Lake Kolon	ACR SCH							1				
352	NA41	2022.07.18	Lake Kolon	ACR SCH							1				
353	NA42	2022.07.18	Lake Kolon	ACR SCH							2				
354	NA43	2022.07.18	Lake Kolon	ACR SCH							2				
355	NA44	2022.07.18	Lake Kolon	ACR SCH							1				
356	NA45	2022.07.18	Lake Kolon	ACR SCH							1				
357	NA46	2022.07.18	Lake Kolon	ACR SCH								1			
358	NA47	2022.07.18	Lake Kolon	ACR SCH								1			
359	NA49	2022.07.19	Lake Kolon	PAR CAE								1			
360	NA51	2022.07.20	Lake Kolon	ACR SCI		1									
361	NA52	2022.07.20	Lake Kolon	ACR SCI								1			
362	NA53	2022.07.21	Lake Kolon	LOC LUS								1			
363	NA54	2022.07.22	Lake Kolon	ACR SCI							1				
364	NA55	2022.07.22	Lake Kolon	ACR SCI								1			
365	NA56	2022.07.23	Lake Kolon	LOC NAE								3			
366	NA57	2022.07.24	Lake Kolon	ACR SCI								1			
367	NA58	2022.07.27	Lake Kolon	LOC LUS								1			
368	NA59	2022.07.27	Lake Kolon	LOC FLU								1			
369	NA60	2022.07.28	Lake Kolon	LUS MEG	1										
370	NA61	2022.07.30	Lake Kolon	LOC LUS								2			
371	NA62	2022.08.02	Lake Kolon	LOC LUS								1			
372	NA63	2022.08.03	Lake Kolon	LOC NAE							1	1			
373	NA64	2022.08.03	Lake Kolon	LOC LUS		1					6				
374	NA65	2022.08.03	Lake Kolon	LOC LUS								1			
375	NA66	2022.08.04	Lake Kolon	LOC LUS								1			
376	NA67	2022.08.08	Lake Kolon	LOC LUS								2			
377	NA69	2022.08.12	Lake Kolon	ACR RIS								1			
378	NA70	2022.08.13	Lake Kolon	LUS LUS	1	4									
379	NA71	2022.08.16	Lake Kolon	ACR ARU								1			
380	NA72	2022.08.16	Lake Kolon	LUS LUS		1									
381	NA74	2022.08.29	Lake Kolon	ERI RUB	1										
382	NA75	2022.09.21	Lake Kolon	ERI RUB		1									
383	NA76	2022.10.05	Lake Kolon	ACR SCI					1						
384	NA77	2022.10.10	Lake Kolon	ERI RUB		1									
385	NA78	2022.11.01	Lake Kolon	TUR MER		1									
386	NA80	2022.11.06	Lake Kolon	TUR MER					1						
387	KG22	2022.03.18	Ócsa	TUR MER		2									
388	KG23	2022.03.18	Ócsa	TUR MER		11									
389	KG24	2022.03.19	Ócsa	PAR MAJ		1									
390	KG25	2022.03.19	Ócsa	PAR MAJ		1									
391	KG26	2022.03.19	Ócsa	PAR MAJ		2									
392	KG27	2022.03.19	Ócsa	TUR MER		2									
393	KG28	2022.03.22	Ócsa	COC COC		1									
394	KG29	2022.03.24	Ócsa	TUR PHI		1									
395	KG30	2022.03.25	Ócsa	ERI RUB		1									
396	KG31	2022.03.25	Ócsa	TUR MER		1									1
397	KG32	2022.04.04	Ócsa	ERI RUB		1									
398	KG34	2022.04.10	Ócsa	TUR MER		5									
399	KG35	2022.04.10	Ócsa	TUR MER		8									
400	KG36	2022.04.10	Ócsa	ERI RUB		5									

	A	B	C	D	E	F	G	H	I	J	K	L	M	N	O
401	KG37	2022.04.11	Ócsa	TRO TRO		1									
402	KG38	2022.04.11	Ócsa	TRO TRO		1									
403	KG39	2022.04.12	Ócsa	ERI RUB		2									
404	KG40	2022.04.13	Ócsa	ERI RUB		2									
405	KG41	2022.04.13	Ócsa	TUR PHI		2									
406	KG42	2022.04.16	Ócsa	ERI RUB		3									
407	KG43	2022.04.16	Ócsa	TUR MER		3									
408	KG44	2022.04.16	Ócsa	TUR MER		1									
409	KG45	2022.04.16	Ócsa	TRO TRO		2									
410	KG46	2022.04.16	Ócsa	TUR MER		3									
411	KG47	2022.04.16	Ócsa	ACR SCH		1									
412	KG48	2022.04.23	Ócsa	TUR MER		1									
413	KG49	2022.04.23	Ócsa	TUR MER		1									
414	KG50	2022.04.23	Ócsa	SYL ATR		1									
415	KG51	2022.04.23	Ócsa	ACR SCH		3									
416	KG52	2022.04.23	Ócsa	SYL COM		1									
417	KG53	2022.04.23	Ócsa	ACR SCH								1			
418	KG54	2022.04.23	Ócsa	ACR SCI				1							
419	KG55	2022.04.30	Ócsa	ACR SCI		1									
420	KG56	2022.05.07	Ócsa	PHY COL		1									
421	KG57	2022.05.07	Ócsa	LUS MEG		2									
422	KG58	2022.05.07	Ócsa	PHY COL		1									
423	KG59	2022.05.07	Ócsa	LOC LUS								1			
424	KG60	2022.05.07	Ócsa	LUS MEG	1	2									
425	KG61	2022.05.15	Ócsa	TUR MER	1	9									
426	KG62	2022.05.15	Ócsa	TUR PHI		1									
427	KG63	2022.05.15	Ócsa	SYL ATR		1									
428	KG65	2022.05.28	Ócsa	PAR MAJ		1									
429	KG66	2022.06.11	Ócsa	TUR PHI		1									
430	KG67	2022.06.11	Ócsa	SIT EUR		1									
431	KG68	2022.06.11	Ócsa	TUR PHI		1						1			
432	KG69	2022.06.12	Ócsa	LUS MEG		2									
433	KG70	2022.06.18	Ócsa	SYL ATR		1									
434	KG71	2022.06.18	Ócsa	SYL ATR		6									
435	KG72	2022.06.18	Ócsa	LOC LUS		1					2	5			
436	KG73	2022.06.18	Ócsa	TRO TRO		1									
437	KG74	2022.06.18	Ócsa	TUR PHI		3						1			
438	KG75	2022.06.18	Ócsa	LOC LUS							17	6			
439	KG76	2022.06.18	Ócsa	LOC LUS		1					21	6			
440	KG77	2022.07.08	Ócsa	ACR RIS		1									
441	KG78	2022.07.08	Ócsa	ACR SCI		1									
442	KG79	2022.07.08	Ócsa	LOC LUS								1			
443	KG80	2022.07.08	Ócsa	LOC LUS							1	2			
444	KG81	2022.07.08	Ócsa	LOC LUS							6	3			
445	KG82	2022.07.08	Ócsa	TUR MER		1									
446	KG83	2022.07.08	Ócsa	LOC LUS								1			
447	KG84	2022.07.08	Ócsa	LOC LUS								2			
448	KG85	2022.07.08	Ócsa	ACR SCH								1			
449	KG86	2022.07.10	Ócsa	LOC LUS								1			
450	KG87	2022.07.10	Ócsa	ACR SCI								1			
451	KG88	2022.07.10	Ócsa	LOC LUS								2			
452	KG89	2022.07.10	Ócsa	LOC LUS							5				
453	KG90	2022.07.10	Ócsa	LOC LUS								1			
454	KG93	2022.07.11	Ócsa	ACR SCI								1			
455	KG94	2022.07.11	Ócsa	ACR SCH								2			
456	KG95	2022.07.13	Ócsa	LOC LUS								2			
457	KG96	2022.07.13	Ócsa	ACR SCH								1			
458	KG97	2022.07.13	Ócsa	ACR SCH								2			
459	KG98	2022.07.16	Ócsa	TUR MER								1			
460	KG99	2022.07.16	Ócsa	ACR MEL		2						1			
461	KG100	2022.07.16	Ócsa	TUR PHI		1									
462	KG101	2022.07.16	Ócsa	LOC LUS		1						1			
463	KG102	2022.07.16	Ócsa	ACR RIS								1			
464	KG103	2022.07.16	Ócsa	ACR MEL							1	1			
465	KG104	2022.07.16	Ócsa	ACR SCI		1									
466	KG109	2022.07.19	Ócsa	ACR RIS								1			
467	KG110	2022.07.20	Ócsa	ACR SCI		1									
468	KG111	2022.07.20	Ócsa	ACR RIS		1									
469	KG112	2022.07.23	Ócsa	ACR SCH								1			
470	KG113	2022.07.23	Ócsa	ACR RIS		2									
471	KG114	2022.07.23	Ócsa	ACR RIS		1						2			
472	KG115	2022.07.23	Ócsa	LOC LUS							2	3			
473	KG116	2022.07.25	Ócsa	LOC LUS		1						4			
474	KG117	2022.07.26	Ócsa	LUS MEG								1			
475	KG118	2022.07.30	Ócsa	ACR RIS								1			
476	KG121	2022.08.06	Ócsa	LOC LUS								1			
477	KG122	2022.08.06	Ócsa	SYL ATR	1										
478	KG124	2022.08.06	Ócsa	SYL COM	1										
479	KG125	2022.08.06	Ócsa	ACR SCI							1	2			
480	KG127	2022.08.14	Ócsa	SYL BOR		1									
481	KG128	2022.08.14	Ócsa	ACR SCI								1			
482	KG129	2022.08.14	Ócsa	LUS LUS		2									
483	KG130	2022.08.15	Ócsa	ERI RUB		1									
484	KG131	2022.08.17	Ócsa	ERI RUB	1										
485	KG132	2022.08.17	Ócsa	LUS LUS	1										
486	KG133	2022.08.17	Ócsa	SYL ATR		1									
487	KG134	2022.08.18	Ócsa	LUS LUS	1										
488	KG135	2022.08.18	Ócsa	SYL ATR	3	1									
489	KG136	2022.08.18	Ócsa	LUS LUS	1										
490	KG137	2022.08.18	Ócsa	LUS LUS		4									
491	KG138	2022.08.19	Ócsa	ERI RUB		1									
492	KG139	2022.08.19	Ócsa	LUS MEG	2	1					1				
493	KG140	2022.08.22	Ócsa	LUS LUS	1										
494	KG141	2022.08.22	Ócsa	SYL BOR		1									
495	KG142	2022.08.23	Ócsa	LUS LUS	2	1									
496	KG143	2022.08.23	Ócsa	ERI RUB	1										
497	KG145	2022.08.23	Ócsa	LUS MEG		1									
498	KG148	2022.08.24	Ócsa	ACR SCI								1			
499	KG152	2022.08.25	Ócsa	ACR RIS		1									
500	KG153	2022.08.25	Ócsa	ERI RUB	1										

	A	B	C	D	E	F	G	H	I	J	K	L	M	N	O
501	KG155	2022.08.25	Ócsa	ACR RIS		1									
502	KG156	2022.08.25	Ócsa	ERI RUB	2										
503	KG157	2022.08.25	Ócsa	SYL COM		1									
504	KG159	2022.08.26	Ócsa	SYL COM		1									
505	KG160	2022.08.26	Ócsa	LUS MEG	1										
506	KG161	2022.08.26	Ócsa	SYL COM		1									
507	KG162	2022.08.26	Ócsa	SYL ATR	1										
508	KG163	2022.08.26	Ócsa	LUS MEG	2										
509	KG164	2022.08.26	Ócsa	SYL ATR	2	1									
510	KG165	2022.08.27	Ócsa	LUS MEG	1										
511	KG166	2022.08.27	Ócsa	ACR SCI		1									
512	KG167	2022.08.28	Ócsa	SYL ATR	1										
513	KG170	2022.09.03	Ócsa	SYL COM		1									
514	KG173	2022.09.08	Ócsa	ERI RUB		1									
515	KG174	2022.09.11	Ócsa	ACR RIS		1									
516	KG175	2022.09.16	Ócsa	TUR MER	1	1									
517	KG176	2022.09.23	Ócsa	SYL ATR		1									
518	KG177	2022.09.23	Ócsa	ERI RUB		1									
519	KG178	2022.09.24	Ócsa	ACR SCH		1									
520	KG179	2022.09.25	Ócsa	ERI RUB	1	1									
521	KG180	2022.09.25	Ócsa	TUR PHI		1									
522	KG181	2022.09.25	Ócsa	SYL ATR		1									
523	KG182	2022.09.25	Ócsa	CER BRA		1									
524	KG183	2022.10.03	Ócsa	ERI RUB		1									
525	KG184	2022.10.08	Ócsa	TUR PHI	1			1			1				
526	KG185	2022.10.08	Ócsa	ERI RUB	1										
527	KG186	2022.10.08	Ócsa	ERI RUB		1									
528	KG187	2022.10.08	Ócsa	FRI COE		1									
529	KG188	2022.10.21	Ócsa	ERI RUB	2	1									
530	KG189	2022.10.22	Ócsa	ERI RUB	1										
531	KG190	2022.10.22	Ócsa	TUR MER		1									
532	KG191	2022.10.22	Ócsa	PAR MAJ		1									
533	KG192	2022.10.22	Ócsa	TUR MER		1									
534	KG193	2022.10.22	Ócsa	TUR MER		3									
535	KG194	2022.10.22	Ócsa	ERI RUB		1									
536	KG195	2022.10.22	Ócsa	ERI RUB			3								
537	KG301	2022.10.27	Ócsa	PAS MON					1						
538	KG302	2022.10.29	Ócsa	ERI RUB		1									
539	KG303	2022.10.29	Ócsa	TUR MER		3									
540	KG304	2022.10.29	Ócsa	TUR MER	1	1									
541	KG305	2022.11.12	Ócsa	TUR MER		1									
542	**Summary**				**241**	**357**	**5**	**4**	**9**	**6**	**105**	**216**	**1**	**11**	**1**
543															
544	**Abbreviations:**														
545	**Iric**	*Ixodes ricinus*													
546	**Ifro**	*Ixodes frontalis*													
547	**Iliv**	*Ixodes lividus*													
548	**Haec**	*Haemaphysalis concinna*													
549	**Hyr**	*Hyalomma rufipes*													
550	**Dret**	*Dermacentor reticulatus*													
551	**L**	Larva													
552	**N**	Nymph													
553	**F**	Female													
554														Total number:	**956**
